# Insights into the microbiota profile of *Pediculus humanus capitis* using metagenomic next-generation sequencing and molecular detection of unexpected pathogen DNA in Hunan Province, China

**DOI:** 10.1186/s13071-026-07471-5

**Published:** 2026-05-27

**Authors:** Yi-Tian Fu, Yuan-Ping Deng, De-Yong Duan, Yan-Yan Peng, Yi-Liu Liu, Ya Zhang, Zi-Kai Xu, Hany M. Elsheikha, Guo-Hua Liu

**Affiliations:** 1 Research Center for Parasites & Vectors, College of Veterinary Medicine, Hunan Agricultural University, Changsha, 410128 Hunan China; 2 College of Animal Science and Technology, Tarim University, Alaer, 843300 China; 3 Faculty of Medicine and Health Sciences, School of Veterinary Medicine and Science, University of Nottingham, Loughborough, LE12 5RD UK

**Keywords:** Head louse, *Pediculus humanus capitis*, Metagenomic next-generation sequencing, Microbiota, Pathogen DNA

## Abstract

**Background:**

The head louse, *Pediculus humanus capitis,* remains a significant public health concern affecting millions of people worldwide and has been implicated as a potential vector for multiple human pathogens. Characterization of the microbiota of head lice could improve our understanding of their public health significance and potential role in pathogen transmission. Here, we characterize the microbiota of  head lice and investigate microbiota differences among different clades of head lice.

**Methods:**

Head lice were collected from Hunan Province, China, and classified into clade A and clade B (CACB) using polymerase chain reaction (PCR)-based genotyping. The microbiota of pooled CACB of head lice samples (*n* = 46) was investigated by metagenomic shotgun sequencing and comparatively analysed at the phylum, genus, and species levels. In addition, the prevalence of potential pathogen DNA in head lice samples (*n* = 204) was assessed using real-time PCR with stringent negative controls.

**Results:**

We obtained non-redundant CACB microbial gene catalog comprising 79,232 genes, of which 4.70% (3,722 genes) were taxonomically assigned. The relative abundance of bacteria (2.52%) was higher than that of eukaryotes (2.04%), viruses (0.11%), and archaea (0.02%). Comparative analysis identified 655 and 750 unique genes in CACB, respectively. The dominant phyla in the CACB of head lice were Proteobacteria. At the genus level, DNA sequences corresponding to *Anaplasma* (25.98%; 53/204), *Mycobacterium* (24.02%; 49/204), *Chlamydia* (23.53%; 48/204), *Ehrlichia* (10.29%; 21/204), and *Vibrio* (0.49%; 1/204) were detected, suggesting the presence of bacterial DNA from these taxa.

**Conclusions:**

Our results provide a preliminary characterization of the annotated fraction of the CACB microbiome in head lice. The high proportion of unannotated genes (>95%) underscores the limited representation of louse-associated microbial genomes in public databases and suggests  substantial, yet unexplored, microbial diversity. The detection of pathogen DNA does not confirm organism viability or vector competence,however it may suggest prior exposure, mechanical carriage, or residual DNA from blood meals. These exploratory findings contribute new insights into the microbiota associated with human lice.

**Graphical Abstract:**

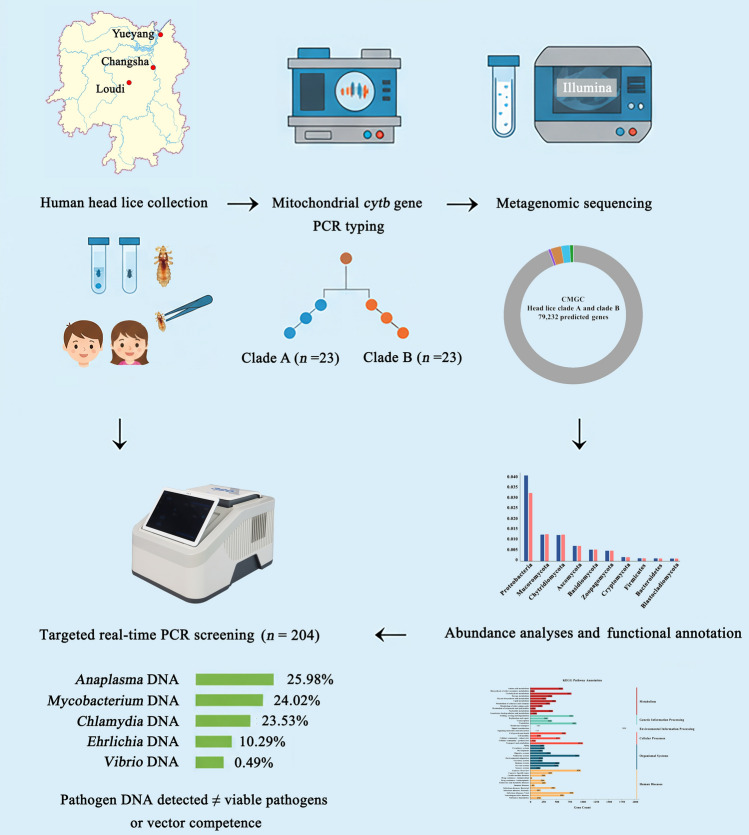

**Supplementary Information:**

The online version contains supplementary material available at 10.1186/s13071-026-07471-5.

## Background

Human lice *Pediculus humanus*, including head lice *P. h. capitis* and body lice *P. h. humanus*, are obligatory ectoparasites. They are among the most frequently detected ectoparasites worldwide [1]. Head lice mainly infest the scalp and hair of the hosts and cause intense scalp itchiness, eczematous dermatitis secondary to hypersensitivity to louse saliva, dermatitis, and cervical/occipital lymphadenopathy [2, 3]. Head lice are not regarded as vectors for infectious agents, while body lice can serve as vectors for the transmission of pathogens to humans, such as *Bartonella quintana* (the causative agent of trench fever), *Borrelia recurrentis* (the causative agent of relapsing fever), and *Rickettsia prowazekii* (the causative agent of epidemic typhus) [3]. However, some recent studies corroborate the vectorial capacity of head lice, which have been considered potential vectors of *B. quintana* [4, 5]. Head lice can also transmit *R. prowazekii* in laboratory environments [6, 7], suggesting that they might play a role in the transmission of bacterial diseases.

In addition, DNA of several pathogens, such as *B. recurrentis*, *Coxiella burnetii*, *Yersinia pestis*, and *Acinetobacter* species, has been detected in head lice [1]. However, these findings are based on conventional polymerase chain reaction (PCR), which is simple and fast but has limited ability to identify novel or unexpected pathogens [8]. The application of culture-independent techniques such as metagenomic next-generation sequencing (mNGS) has enabled rapid and comprehensive characterization of microbial communities and has been extensively used for metagenomic and microbiome investigation [9]. Notably, mNGS-based approaches are increasingly recognized as valuable tools within the One Health framework, which emphasizes the interconnectedness of human, animal, and environmental health. Ectoparasites, such as lice, play a critical role at the human–environment interface, and their associated microbiota may reflect pathogen transmission dynamics [10]. By enabling the simultaneous detection of bacteria, viruses, fungi, and parasites without prior assumptions regarding target organisms, mNGS facilitates integrated surveillance strategies that are essential for identifying emerging and neglected vector-borne pathogens [11, 12].

Despite the rapid development of microbiota research, the number of studies on louse microbiota remains limited, with only two studies available to date. One study compared the gut microbiota of 16 head lice and 7 body lice [13]. Another study analyzed the gut microbiota of 6 female and 3 male head lice, identifying a total of 223 bacterial genera [2]. However, both studies relied solely on 16S rRNA gene amplicon sequencing and were limited by small sample sizes, making it difficult for their findings to comprehensively and accurately reflect the true composition of the head lice gut microbiota. Phylogenetic analyses based on the mitochondrial cytochrome *b* (mt *cytb*) gene grouped human lice into six clades, namely A to F [1]. The differences in the microbiome among those clades are still unknown. Therefore, it is necessary to conduct in-depth studies utilizing more advanced metagenomic technologies to more comprehensively elucidate the characteristics and differences of the microbiomes across different genetic clades of head lice, and to systematically assess their potential risk of harboring pathogens.

In this study, we employed mNGS to investigate the differences in the microbiota profiles between two different head lice clade A and clade B (CACB) from Hunan Province, China, and detected the presence of potential pathogen DNA within head lice samples.

## Methods

### Sample collection, preservation, and DNA extraction

A total of 240 schoolchildren from Yueyang City, Hunan Province, China, were examined for head lice, with a prevalence of 5.83% (14/240). Adult head lice (*n* = 46) were collected from 14 infested schoolchildren. Live head lice were physically collected from the hair and scalps of the infested schoolchildren using sterile fine-toothed lice combs and sterile forceps [14]. Immediately after removal, the collected lice were placed into sterile 1.5-ml Eppendorf tubes. These tubes were then temporarily stored in a portable cooler with ice packs and transported to the laboratory within 2–4 h. All lice were washed with normal saline to remove the human hair and exuvium and then stored in 70% ethyl alcohol (v/v) at −40 ℃ until further processing. To minimize external contamination, we used strict sterile techniques. Before DNA extraction, all instruments and solutions were sterilized by ultraviolet (UV) treatment for 12 h and autoclaving. All procedures were performed inside a biosafety cabinet sterilized by UV to avoid sample contamination. The extraction of DNA was performed according to the manufacturer’s instructions provided with the Promega Kit (Wizard^®^ SV Genomic DNA Purification System, USA).

### PCR and preliminary phylogenetic analysis

We used PCR to amplify the partial sequence of the mt *cytb* gene and assign head lice to clade A and clade B (CACB). CytbF1 (5′-GAGCGACTGTAATTACTAATC-3′) and CytbR1 (5′-CAACAAAATTATCCGGGTCC-3′) were used as primers [15]. The amplification conditions included incubation at 95 ℃ for 15 min, followed by 40 cycles of 95 ℃ for 1 min, 56 ℃ for 30 s, and 72 ℃ for 1 min, then a final extension at 72 ℃ for 5 min. The PCR products were analyzed by 1.5% agarose gel electrophoresis and sequenced using the Sanger method by Tsingke Biotechnology Co. Ltd. (Hunan, China). The obtained sequences (about 320 bp) were aligned together with available mt *cytb* gene data of human lice by using Clustal 2.0 [16]. Based on MEGA v12.0 [17], the aligned sequences were used to construct a neighbor-joining (NJ) tree with 1000 bootstrap replications. Samples were then grouped, labeled as CACB, and submitted to Novogene Bioinformatics Technology Co. Ltd. (Tianjin, China) for metagenomic analysis.

### Library construction and metagenome sequencing

The extracted DNA samples were used for metagenome sequencing. A total amount of 2.65 µg for clade A (23 samples) and 1.82 µg for clade B (23 samples) were used as the primary material for DNA preparations. For each clade individually, following the instruction of NEBNext^®^ Ultra^™^ DNA Library Prep Kit for Illumina (NEB, USA), sequencing libraries were constructed with unique index codes, and then pooled for sequencing on the Illumina HiSeq platform. Using Covaris ultrasonic crusher, the DNA samples were sonicated into a size of 350 bp, and DNA fragments were then end-polished, A-tailed, and ligated with a full-length adaptor Illumina sequencing and PCR amplification. The AMPure XP system and Agilent2100 Bioanalyzer were used to purify PCR products and analyze the size distribution of the libraries, and real-time PCR (RT-PCR) was used to quantify the PCR products. The grouping of the index-coded samples was performed by a cBot Cluster Generation System according to the manufacturer’s instructions. After library preparation, the samples were sequenced on the Illumina HiSeq platform, generating paired-end reads.

### Preprocessing of the raw sequence data

A total of 9630.29 Mbp high-quality reads were obtained by filtering out low-quality reads, reads with adapter or ambiguous “N” bases, and reads from host origin (head lice genome) from the raw data (9656.22 Mbp) with an effective rate of 99.7%. Clean reads that did not match, or showed low similarity to sequences in the nonredundant (NR) database, were discarded. The dataset was analyzed based on Bowtie v.2.2.4 with parameters “–end-to-end, –sensitive, -I 200, -X 400” [18]. The clean reads were then assembled to contigs using MEGAHIT v. 1.2.9 with the following parameters “–k-min 35 –k-max 95 –k-step 20 -min-contig-len 500 -m 0.1” [19]. Clean data were compared with assembled contigs based on Bowtie v.2.2.4 [18], using the above-mentioned parameters. The unused reads of CACB were subsequently assembled using the same software and parameters.

### Gene prediction and abundance analyses

Open reading frames (ORFs) were predicted from the obtained contigs (≥ 500 bp) by Meta Gene Mark v. 2.10, and ORFs shorter than 100 nt were removed [20–22]. A unique catalog with NR genes was created using CD-HIT v.4.5.8 with the parameters “-c 0.95, -G 0, -aS 0.9, -g 1, -d 0” [23], with a coverage cutoff of 0.90 and sequence identity of 0.95. The reads were aligned within the gene catalog to determine the gene abundances by Bowtie v.2.2.4 and the parameters were set as: –end-to-end, –sensitive, -I 200, -X 400. To reduce the error rate, genes with fewer than two reads were filtered out, and only those with at least two reads were considered present in a sample [24, 25]. The statistical abundance of each sample was calculated based on the gene length and the number of mapped reads [26].

### Species annotation

All predicted genes were aligned to the NR database for taxonomic annotations, such as archaea, bacteria, eukaryotes, and viruses, using DIAMOND v.0.9.9 with the default parameters. The results of each gene obtained as previously described were preserved for distinguishing the taxonomic groups. The gene levels of the taxonomy (from kingdom to species) were recognized based on the lowest common ancestor (LCA) algorithm with the software MEGAN [27]. The abundances of each annotated gene were used to calculate the abundances of each taxonomical hierarchy group and produce the Krona analysis.

### Functional annotations

The constructed NR gene catalog was aligned to the functional databases, including the CAZy database (v.201801, http://www.cazy.org/), EggNOG database (v.4.5, http://eggnogdb.embl.de/#/app/home), and KEGG database (v.2018–01-01, http://www.kegg.jp/kegg/) [28, 29], using DIAMOND v.0.9.9 [30] with default parameters setting of blastp “-e 1e-5”. To quantify the functional profiles, sequences assigned to these databases with the highest Blast Hit were used to calculate the relative abundance of different functional hierarchies and pathways [31].

### Molecular detection of pathogen DNA

The metagenomic sequencing analysis (*n* = 46) enabled the initial identification of potential pathogen DNA in head lice. To validate these metagenomic findings and to estimate the prevalence of the identified pathogen DNA across a broader geographic range in Hunan Province, we performed targeted real-time (RT)-PCR screening using an independent and larger sample set of head lice (*n* = 204). This additional batch was collected from the cities of Yueyang (*n* = 76), Loudi (*n* = 101), and Changsha (*n* = 27), Hunan Province, China. The collections procedures were the same as above.

The reaction was performed using 10 µl 2X SYBR Green *Pro Taq* HS Premix^*6^ (Accurate, Guangdong, China), 0.4 µl ROX reference dye, 0.5 µl of forward/reverse primer, and 2 µl template DNA, adding RNase-free water to 20 µl. The amplification was performed on ABI StepOne^™^ (Thermo Fisher, USA) and involved one cycle of predenaturation at 95 ℃ for 30 s, followed by 40 cycles of 95 ℃ for 5 s and 60 ℃ for 30 s. Strict negative controls (nuclease-free water) were included in each PCR run to rule out reagent contamination. In accordance with the MIQE guidelines [32, 33], the quantification cycle (Cq) terminology and reporting standards were adopted. For our specific assays, the analytical threshold for positivity was empirically defined: a PCR run was considered valid only if the negative control yielded a Cq > 40 and the positive control yielded a Cq < 35. Under these validated conditions, experimental samples exhibiting a Cq < 35 were deemed positive.

### Data analysis

Due to the low DNA yield per individual louse, DNA from 23 lice of each clade was pooled into a single metagenomic library, resulting in one pooled sample per clade. This pooling strategy precludes biological replication and formal statistical comparison between clades. Therefore, all comparisons between CACB were performed using only descriptive statistics. No formal statistical hypothesis testing (e.g., differential abundance testing or multivariate analysis) was applied to the metagenomic profiles.

## Results

### Genotypes and statistics of the head lice

Phylogenetic analysis based on the NJ method and mt *cytb* gene sequences showed that 46 head lice samples were grouped into clades A (*n* = 23) and B (*n* = 23) (Fig. S1). A total of 9630.29 (99.73%) of Mbp clean data was extracted from the total raw data (9,656.22 Mbp), including 4973.23 Mbp in clade A and 4657.06 in clade B. The sequence quality was high, as indicated by 93.0% Q20 and 84.9% Q30. The specific statistical data are presented in Table S1.

### Metagenomic analysis and microorganism overview

**Fig. 1:**
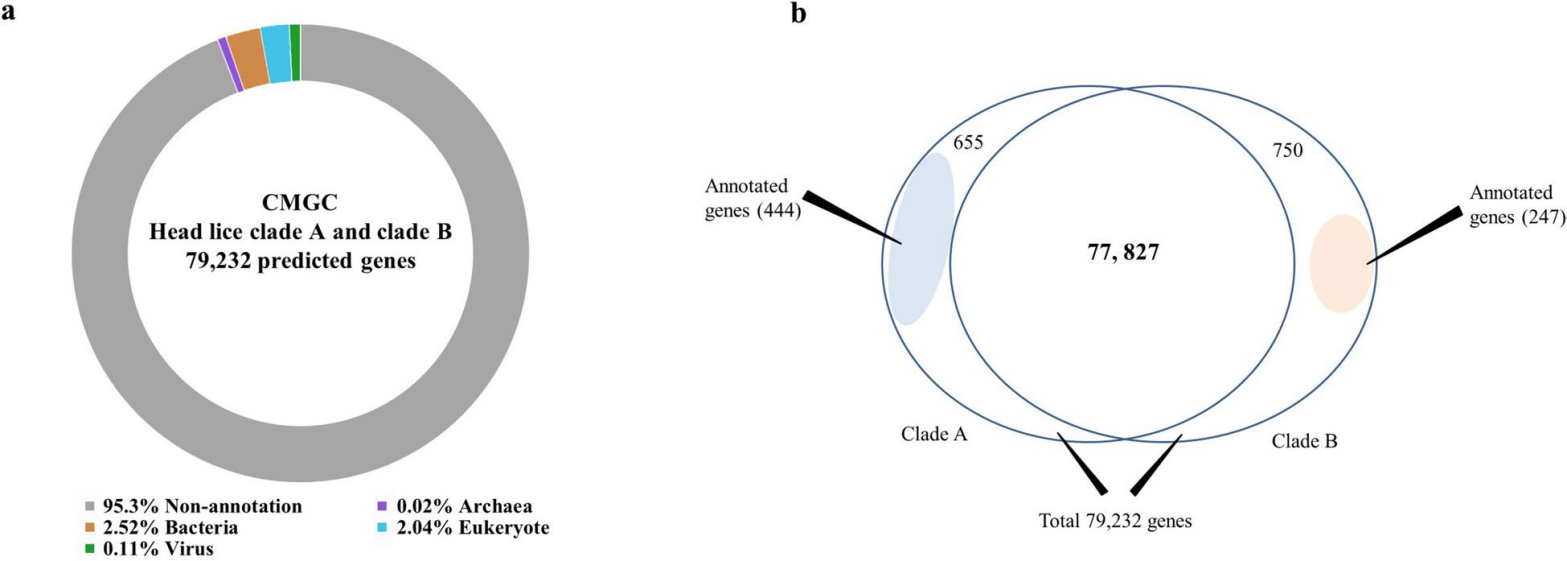
**a** Overview of the predicted and annotated genes in CACB, and **b** analysis of the number of unique genes of clade A and B of *Pediculus humanus capitis*. Each circle represents one sample; the overlapping area represents the shared gene number between two samples; the non-overlapping circle represents unique genes in each sample

Metagenomic sequencing produced a total of 22.8 Gb of raw data. After quality control and removing the host-related raw data, 21.9 Gb of clean data was obtained for subsequent analysis and generated 33,154,890 clean reads in clade A and 31,047,092 clean reads in clade B. According to the metagenomic assembly and ORF prediction, we obtained ORFs of 26.0 Mbp, with 70% of the detected genes recognized as complete. After clustering at 90% nucleotide sequence coverage and 95% sequence identity, we obtained the nonredundant CACB microbial gene catalog (CMGC) with 79,232 genes ranging from 100 bp to 1600 bp (average length 263 bp). However, based on the available databases, only 4.70% (3722/79,232) of the genes in the CMGC were taxonomically identified as archaea (0.02%), bacteria (2.52%), eukaryotes (2.04%), and viruses (0.11%) (Fig. 1a). The rest, 95.30%, of the genes were not annotated, suggesting the presence of many unknown taxonomic species in the CACB, which may be due to the limited representation of louse-associated symbiont genomes in public databases. There were also unique genes detected in the head lice clade A (*n* = 655) and clade B (*n* = 750), with 77,827 shared genes (Fig. 1b). However, in the unique genes, only 67.79% (444/655) in clade A and 32.93% (247/750) in clade B were annotated at the species level.

The genes from archaea, bacteria, eukaryotes, and viruses were assigned to 298 families, including 9 archaea families, 153 bacterial families, 124 eukaryote families, and 12 viral families. After the consideration of dietary habits and host traits, the remaining families were attributed to 78 families that infected only animals, including 49 bacterial families, 21 eukaryote families, and 8 viral families.

### Relative abundance of microorganisms

**Fig. 2:**
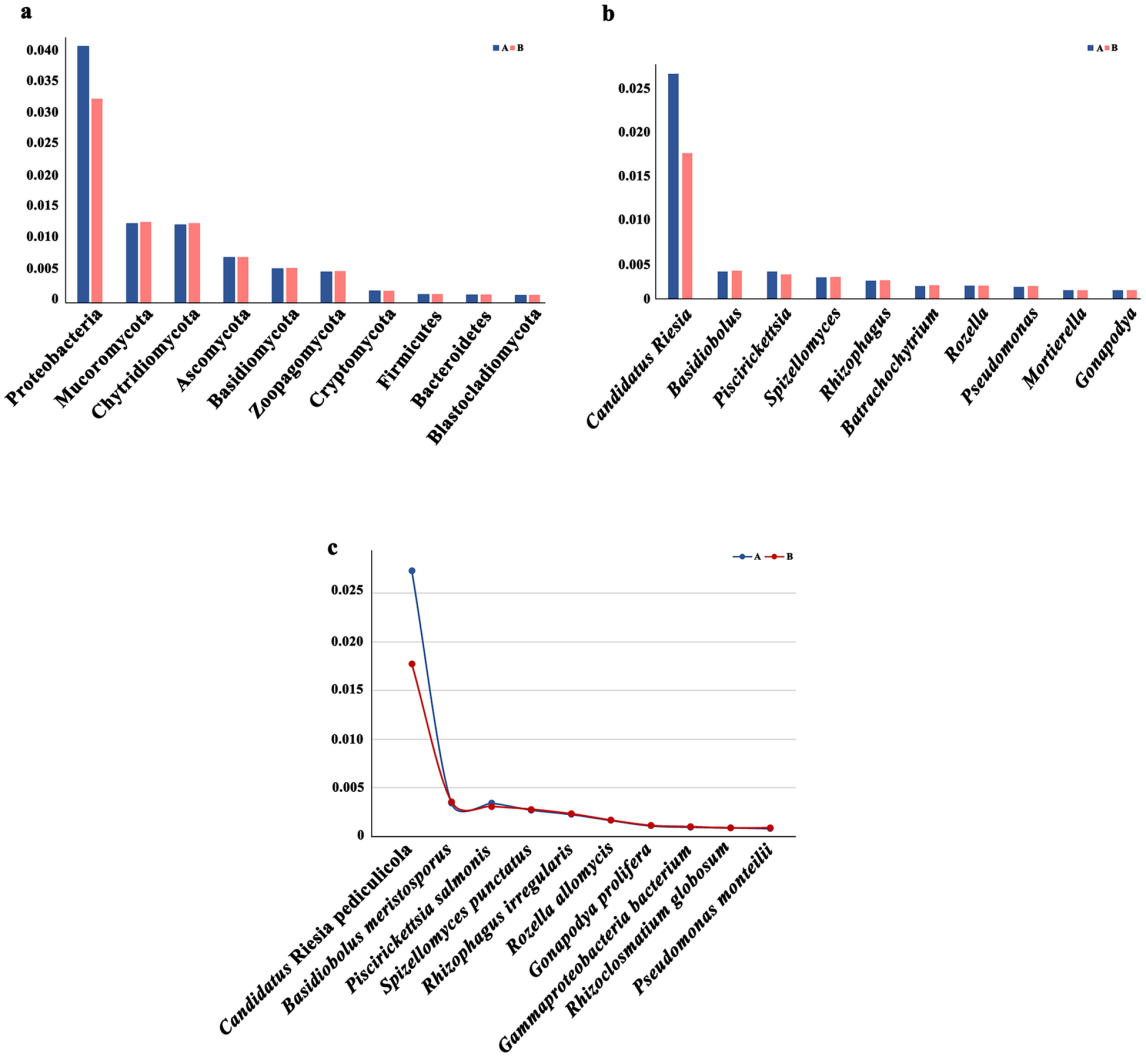
The microbial population characteristics of the ten most abundant species, genera, and phyla in CACB of *Pediculus humanus capitis*. The top ten species (**a**), genera (**b**), and phyla (**c**) are shown. The vertical axis represents the relative abundance of each species/genus/phylum. Comparisons shown are descriptive; statistical significance was not tested

The top ten phyla are shown in Fig. 2a, and the full list of taxa is presented in Table S2. The phylum Proteobacteria was dominant in the CACB (4.35% in clade A and 3.46% in clade B), followed by phyla Mucoromycota, Chytridiomycota, and Ascomycota (Fig. 2a). The genera *Candidatus* Riesa, *Basidiobolus*, *Piscirickettsia*, *Spizellomyces*, *Rhizophagus*, *Batrachochytrium*, *Rozella*, *Pseudomonas*, *Mortierella*, and *Gonapodya* were abundant among CACB (Fig. 2b). *Staphylococcus* was found in the CACB with 0.05% of all identified genes, but the *Streptococcus* gene was not detected in the present study.

Lice-associated pathogens, *Rickettsia*, *Yersinia*, *Serratia*, and *Coxiella*, were identified in the CACB. Other pathogenic genera, such as *Anaplasma*, *Ehrlichia*, *Mycobacterium*, *Chlamydia*, *Salmonella*, and *Vibrio*, were also identified in the CACB with 0.05%, 0.02%, 0.08%, 0.05%, 0.02%, and 0.16% genes, respectively. *Coxiella* was identified in clade A with low abundance but was not shown in clade B. However, *Borrelia* and *Bartonella* species were not detected in CACB in the present study, although they were frequently detected in previous studies.

At the species level, a total of 734 species were obtained and identified in the CACB of head lice. Among these, bacteria and eukaryotes accounted for 472 and 215 species, respectively. However, the viral and archaeal species were less frequently recognized, with only 39 and 17 species, respectively, detected in the CACB. The list of the identified species is presented in Tables S3–S6. As the primary endosymbiont in human lice, *Candidatus* Riesa pediculicola was the dominant species among the ten most abundant species in the CACB, accounting for 2.27% of total bacteria. The top ten species are shown in Fig. 2c. Bacteria species were the most frequently identified. Notably, the average relative abundance of assigned eukaryotic sequences appeared higher than that of bacteria. This observation is unexpected in light of the established literature on the microbiota of *P. h. capitis*, in which bacterial endosymbionts such as Candidatus *Riesia* and environmental taxa such as *Acinetobacter* consistently account for the overwhelming majority of sequencing reads. Given the relatively modest metagenomic sample size and the absence of sequencing negative controls in our study, we cannot rule out the possibility that this unexpected pattern is a dataset-specific artifact driven by methodological or sampling effects (e.g., environmental eukaryotic contamination). As one of the opportunistic human pathogens, *Pseudomonas* species were frequently detected in CACB, with 1.83% of all recognized genes.

### Gene function prediction

A total of 17.23% (*n* = 13,655), 38.40% (*n* = 30,428), and 3.13% (*n* = 2,480) genes were annotated to the KEGG orthologous groups (KOs), the cluster of orthologous groups of protein (COGs), and carbohydrate-active enzymes (CAZymes), respectively. Glycosyl transferases (GTs) were dominant enzymes in the CMGC (cyclin-dependent kinase [CDK], mitogen-activated protein kinase [MAPK], glycogen synthase kinase [GSK3], CDC-like kinase [CLK]) group of protein kinases based on the CAZy database, followed by carbohydrate-binding modules (CBMs), glycoside hydrolases (GHs), auxiliary activities (AAs), and carbohydrate esterases (CEs); those results were similar in the two head lice clades.

**Fig. 3:**
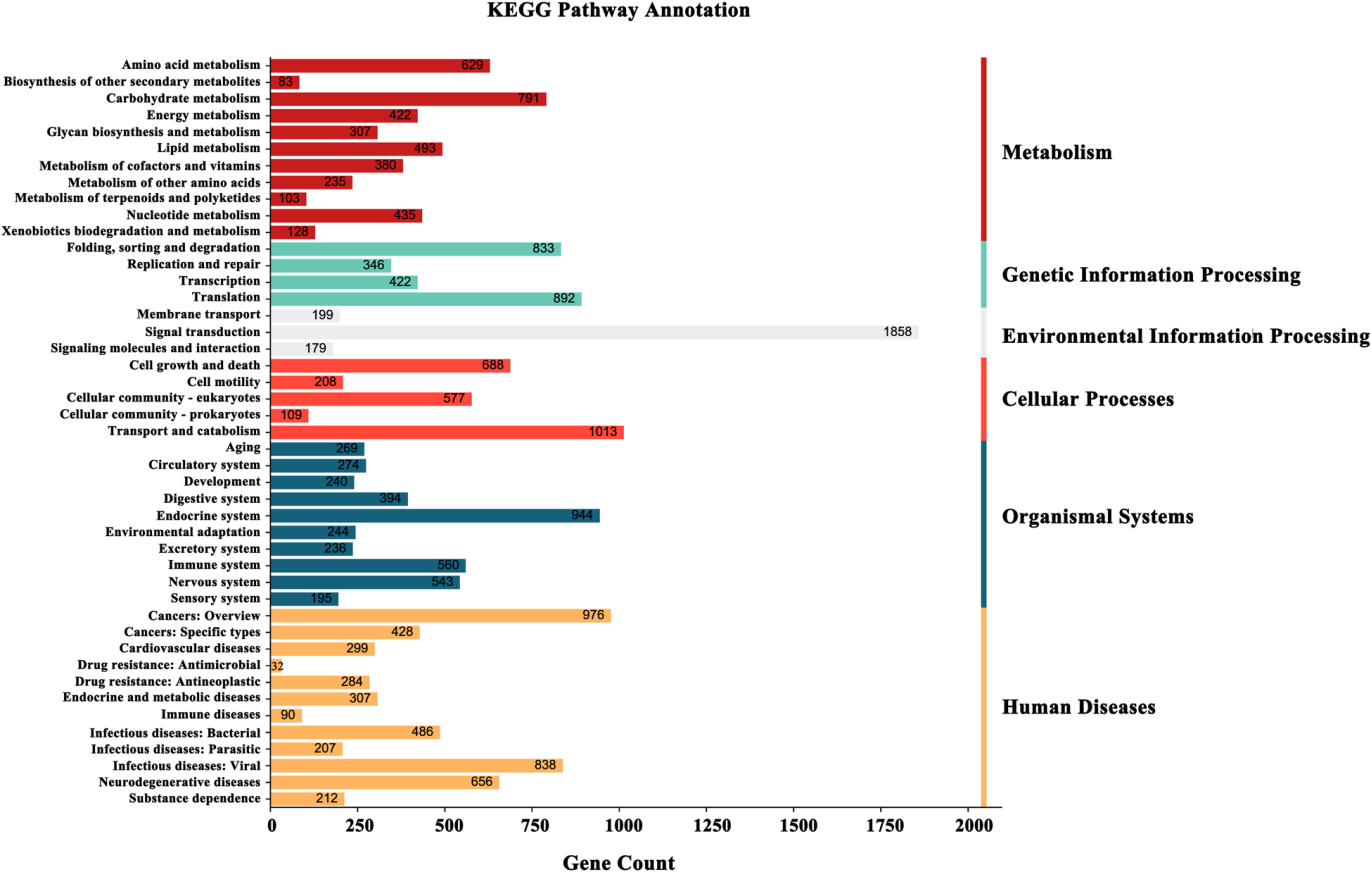
Annotation of Kyoto Encyclopedia of Genes and Genomes (KEGG) pathways related to the number of genes in clades A and B of *Pediculus humanus capitis* at level 1. The black color on the ordinate indicates KEGG level 1, the *y*-axis shows the specific pathway at this level, and the abscissa denotes the number of genes in the pathway. Comparisons shown are descriptive; statistical significance was not tested

**Fig. 4:**
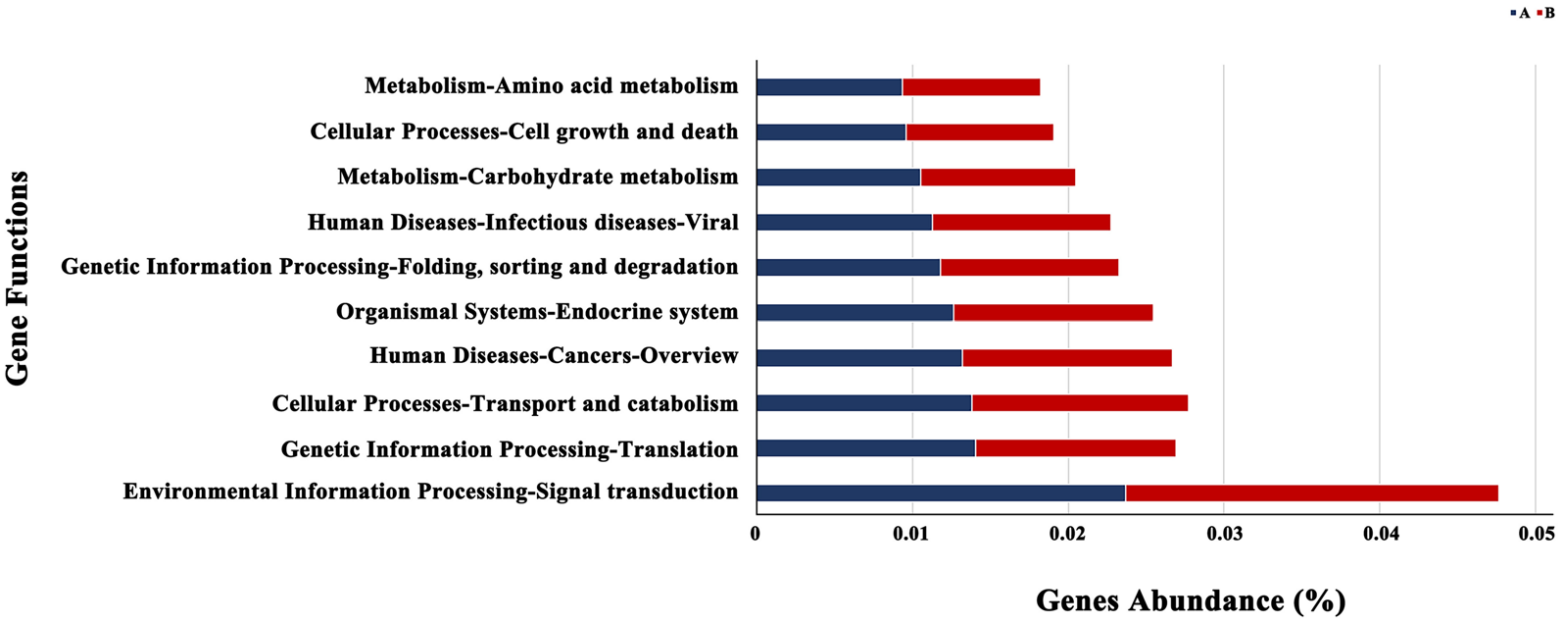
The top ten gene functions of level 2 based on the KEGG database. Comparisons shown are descriptive; statistical significance was not tested

**Fig. 5:**
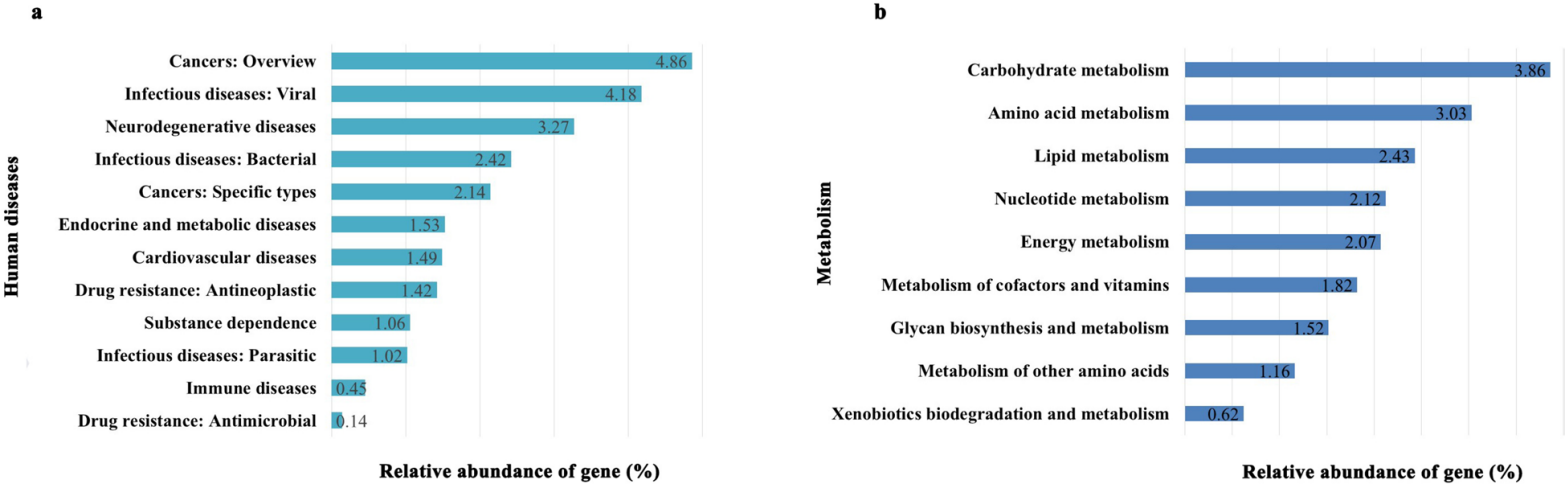
**a** The relative abundance of the microbial genes involved in human diseases and detected in CACB of *Pediculus humanus capitis* at level 2 in KEGG pathway annotation. **b** The relative abundance of the microbial genes involved in metabolism in CACB of *Pediculus humanus capitis* at level 2 in KEGG pathway annotation. Comparisons shown are descriptive; statistical significance was not tested

Using DIAMOND, unique genes were functionally annotated on the basis of the KEGG database. At the level 1 pathway, genes were assigned to metabolism-related genes (5.06%), human diseases (6.08%), genetic information processing (3.15%), organismal systems (4.92%), cellular processes (3.27%), and environmental information processing (2.82%). The gene number of the detailed functions and the top ten pathways of level 2 are shown in Figs. 3 and 4, respectively. The functions were mostly related to human diseases, metabolism, and organismal systems, which contained 12 pathways, 11 pathways, and 10 pathways, respectively. Genes related to cancers (7.0%) and infectious diseases (7.6%) were dominant in human diseases (Fig. 5a), reflecting homology to genes involved in these pathways. The metabolisms of carbohydrate (3.9%), amino acid (3.0%), and lipid (2.4%) were the main processes in the CACB (Fig. 5b), and endocrine (4.7%) was the main process in the organismal system (Fig. S2).

### Molecular detection of pathogen DNA

The DNA sequences belonging to potential pathogens in CACB with low abundance levels from metagenomics were molecularly detected, including *Anaplasma*, *Mycobacterium*, *Chlamydia*, *Ehrlichia*, and *Vibrio* species. The most frequently detected DNA in the samples was from *A. phagocytophilum* (53, 25.98%). DNA of *M. kyorinense* was detected in 24.02% (49/204), *C. trachomatis* was in 23.53% (48/204), and *Ehrlichia* was in 10.29% (21/204). DNA of *Vibrio* species were also detected in only one sample (0.49%), consistent with potential environmental contamination rather than true biological association.

## Discussion

In this study, we used a metagenomic approach to profile and characterize the microbiota of CACB in head lice from China. A substantial proportion (> 95%) of the predicted genes in our dataset could not be taxonomically annotated. This low annotation rate is a common challenge in metagenomic studies of non-model organism microbiomes and likely reflects the scarcity of reference genomes for louse-specific symbionts in public databases. It highlights a substantial reference database bias against non-model organism symbionts. Consequently, our characterization reflects the annotated fraction (4.70%), and the ecological or functional relevance of the vast unclassified genetic repertoire (95.30%) remains unknown. Comparative analysis revealed 655 and 750 unique genes in CACB, respectively. However, the clade comparison in this study is inherently limited by the pooled sequencing design, which provides only a single observation per clade. In the absence of biological replication and statistical testing, these differences may reflect stochastic variation or technical artifacts rather than true biological differences between clades.

Proteobacteria species are well known and the most abundant species in head lice [34–36]. In agreement, our data showed that the phylum Proteobacteria had the highest abundance in CACB (Fig. 2a). These results suggest that the phylum Proteobacteria may contain considerable diversity in *P. h. capitis*. Among the top ten most abundant genera in CACB, *Candidatus* Riesia is the well-established primary obligate endosymbiont of human lice [37]. While *Pseudomonas* has also been previously detected in head lice microbiomes [2], it is not recognized as a common louse endosymbiont and should be interpreted cautiously, as it may reflect environmental or laboratory contamination. By contrast, other eight most abundant genera (*Basidiobolus*, *Piscirickettsia*, *Spizellomyces*, *Rhizophagus*, *Batrachochytrium*, *Rozella*, *Mortierella*, and *Gonapodya*) have never been recorded in head lice. *Acinetobacter* species are frequently reported in head lice from different regions [35, 38]. In our study, *Acinetobacter* species such as *A. baumannii* were also identified in CACB with relatively high abundance. The causative agent of plague, *Y. pestis*, has been reported in *P. h. capitis* in highly plague-endemic regions [38, 39]. By contrast, the present study did not find *Y. pestis,* but *Y*. *kristensenii* was identified with low abundance. It is noteworthy that no *Bartonella*, *Borrelia*, or *Rickettsia* species were identified in CACB in China, although their DNA is frequently detected in head lice and mainly prevalent in Africa, Europe, and America [24, 40], suggesting that the prevalence of those bacterial agents may be geography-specific. Geographic variation in the prevalence of lice-borne pathogens is well documented and may reflect differences in the local reservoir host populations, hygiene conditions, and socioeconomic factors that influence human–lice contact intensity [41, 42]. The absence of *Rickettsia* likely exhibits differences in transmission dynamics and ecology [7, 43].

A total of 46,563 genes were annotated to the KOs, COGs, and CAZymes, with 32,669 unknown genes that were not annotated to any specific functions. It has previously been estimated that the vast majority of microbes within different environments are uncultured with numerous unknown genes and functions [44, 45]. It is important to note that the assignment of genes to KEGG “Human Diseases” categories (e.g., cancer, infectious diseases) reflects sequence homology to genes in those database pathways, not evidence that the lice microbiota themselves cause such diseases. These annotations highlight metabolic or functional modules present in the microbial community that are genetically similar to those studied in human disease contexts.

The prevalence of DNAs of *Mycobacterium*, *Anaplasma*, *Chlamydia*, *Ehrlichia*, and *Vibrio* was determined in 204 head lice. DNA of *A. phagocytophilum* (25.98%, 53/204) was detected in head lice. Clinical cases of *M. kyorinense* have never been reported in China; however, we found DNA of this species in the head lice. In the present study, we detected the DNA of *C. trachomatis* (23.53%; 48/204) in head lice. We also found *Ehrlichia* spp. (10.29%; 21/204) in this study. Three *Vibrio* species (*V. cholerae*, *V. parahaemolyticus*, and *V. vulnificus*) were recognized in CACB, but only one positive sample was detected in 204 head lice. It is crucial to distinguish between the presence of DNA and active infection or vector competence. Specifically, detection of bacterial DNA via mNGS or PCR does not prove the bacteria are viable, nor does it confirm that head lice can biologically transmit these bacterial agents to humans. These findings should be interpreted solely as molecular evidence of DNA presence. The possibility of environmental contamination, residual nucleic acids from human blood meals, or transient mechanical carriage cannot be excluded. No experimental data are available to demonstrate that head lice can biologically replicate or transmit these bacterial agents. Therefore, these molecular detections should be considered hypothesis-generating findings that warrant further investigation through vector competence studies.

As a mainstream tool for studying microbes within animals and environments, mNGS can provide a method to characterize, and quantify microbes and reveal novel genes and pathways. Previous molecular studies on *P. h. capitis* have primarily focused on mitochondrial clade classification, phylogenetic relationships, and the detection of insecticide resistance mutations [42, 46, 47]. While targeted PCR-based approaches have also been employed to screen for specific bacterial pathogens [38, 48], these methods are inherently limited to preselected targets and cannot capture the broader microbial community or its functional potential. By contrast, mNGS enables simultaneous, unbiased characterization of the entire microbial community, including bacteria, eukaryotes, and viruses, along with their associated functional gene repertoires [31]. To the best of our knowledge, the present study represents one of the first applications of shotgun metagenomic sequencing to comprehensively profile both the taxonomic composition and functional potential of the *P. h. capitis* microbiome. Although the mNGS workflow lacked sequencing negative controls, making it difficult to completely exclude background contamination for low-abundance taxa such as *Vibrio* spp., it still served as a valuable exploratory screening tool.

## Conclusions

Using metagenomic sequencing, we profiled the genetic differences in the microbial communities between CACB head lice, focusing on the taxonomically annotated fraction (4.70%). Our data, supported by RT-PCR with negative controls, demonstrated the presence of DNA of *M. kyorinense*, *A*. *phagocytophilum*, and *C. trachomatis* in head lice. These findings do not constitute evidence of active infection or vector competence; rather, they may reflect environmental contamination, blood meal residues, or mechanical carriage. Further experimental work, including bacterial viability assays and transmission studies, is required to determine the biological significance of these findings.

This study has two methodological limitations. First, the modest metagenomic sample size (*n* = 46) limits the statistical power for comparative analyses and the detection of rare taxa. Consequently, the descriptive differences between clades should be interpreted as preliminary insights, necessitating validation in future studies with larger sample sizes. Second, the absence of sequencing negative controls represents a major methodological limitation, as it particularly compromises the ability to rule out contamination from low-abundance environmental organisms, such as certain *Vibrio* spp., in these low-biomass samples. While our subsequent PCR validation on a larger cohort (*n* = 204) with negative controls confirms the presence of pathogen DNA, the overall metagenomic profile, particularly of low-read-count taxa, should be considered preliminary. Future studies with larger sample sizes and stringent contamination controls are needed for definitive characterization.

## Supplementary Information

Supplementary Material 1. Fig. S1 The phylogenetic analysis of 46 head lice samples based on the neighbor-joining (NJ) method.

Supplementary Material 2. Fig. S2 The relative abundance of the microbial genes involved in the organismal system in CACB of *Pediculus humanus capitis* at level 2 in KEGG pathway annotation. Comparisons shown are descriptive; statistical significance was not tested.

Supplementary Material 3. Table S1 Quality control results of the metagenomic DNA of each sample. Table S2 The total phyla predicted in CACB. Table S3 Relative abundance of bacteria species of CACB of *Pediculus humanus capitis*. Table S4 Relative abundance of eukaryote species of CACB of *Pediculus humanus capitis*. Table S5 Relative abundance of 39 viral species of clade A and clade B of *Pediculus humanus capitis.* Table S6 Relative abundance of 17 archaea species of clade A and clade B of *Pediculus humanus capitis*.

## Data Availability

The raw sequence data are available in the Sequence Read Archive (SRA) of the US National Center for Biotechnology Information (NCBI) under BioProject PRJNA817842.

## Abbreviations

PCR: Polymerase chain reaction
mNGS: Metagenomic next-generation sequencing
mt: Mitochondrial
*cytb*: Cytochrome *b*
CACB: Clade A and clade B
RT-PCR: Real-time PCR
ORF: Open reading frame
NR: Nonredundant
LCA: Lowest common ancestor
NJ: Neighbor-joining
CMGC: CACB microbial gene catalog
KO: KEGG orthologous group
COG: The cluster of orthologous groups of protein
CAZyme: Carbohydrate-active enzyme
GT: Glycosyl transferase
CDK: Cyclin-dependent kinase
MAPK: Mitogen-activated protein kinase
GSK3: Glycogen synthase kinase
CLK: CDC-like kinase
CBM: Carbohydrate-binding module
GH: Glycoside hydrolase
AA: Auxiliary activity
CE: Carbohydrate esterase
Cq: Quantification cycle
