# Supplementary figures and images for: Insights into the microbiota profile of Pediculus humanus capitis using metagenomic next-generation sequencing and molecular detection of unexpected pathogen DNA in Hunan Province, China
Source: Parasit Vectors. 2026 May 27;19:298. doi: 10.1186/s13071-026-07471-5 (PMC13397650; doi:10.1186/s13071-026-07471-5)

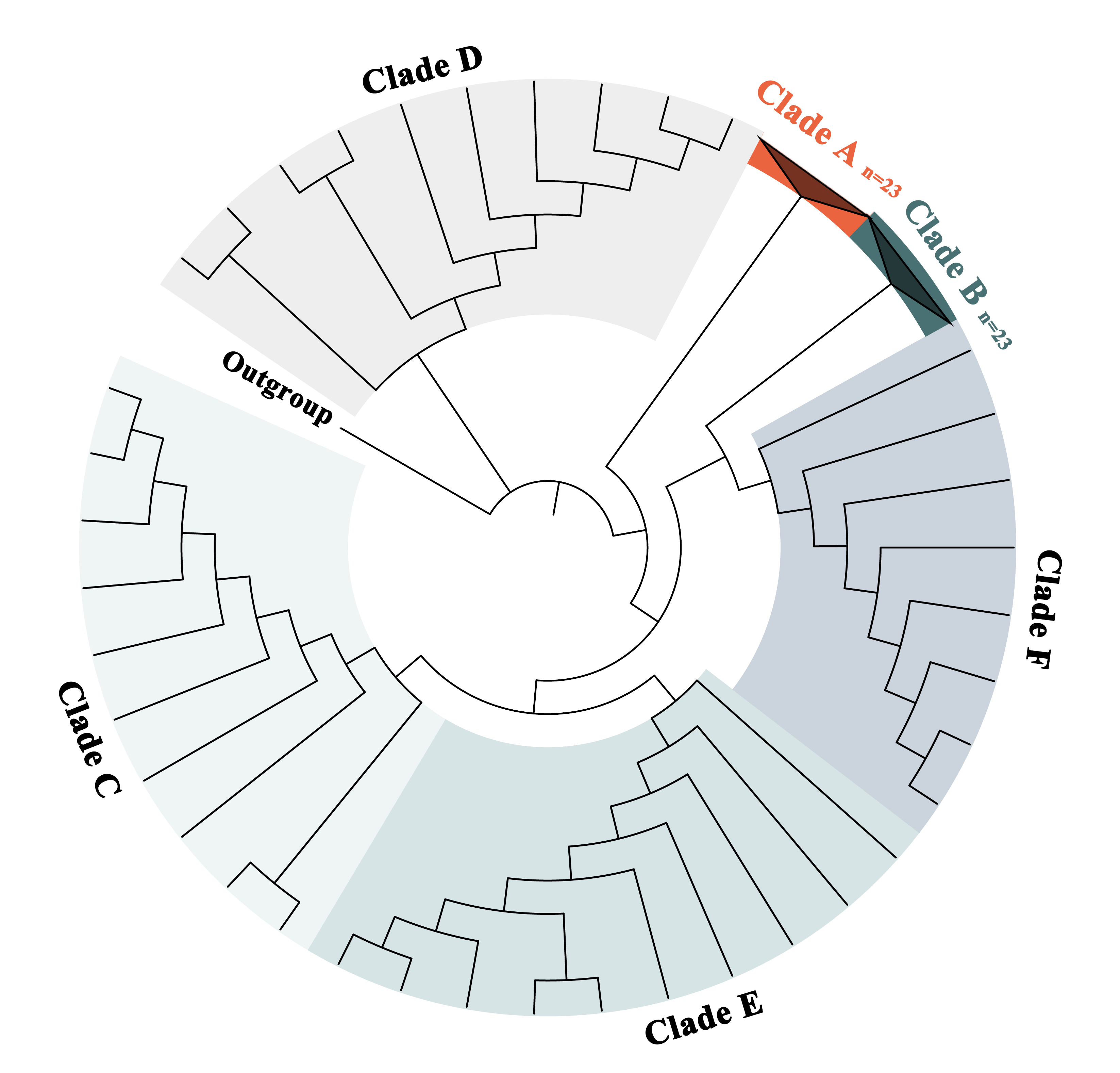

Supplement: Supplementary file 1 — Supplementary Material 1. Fig. S1 The phylogenetic analysis of 46 head lice samples based on the neighbor-joining (NJ) method. [file 13071_2026_7471_MOESM1_ESM.tif]

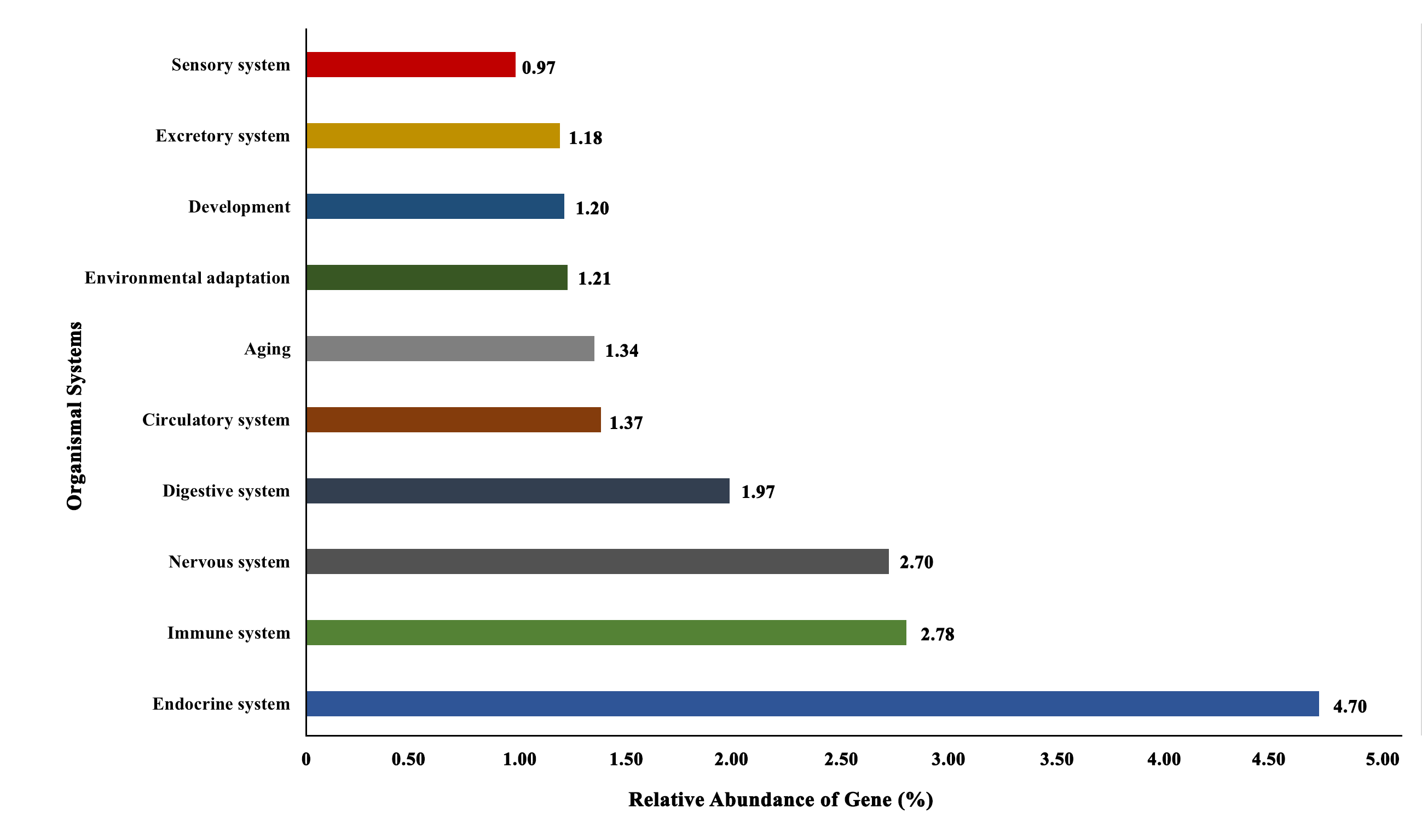

Supplement: Supplementary file 2 — Supplementary Material 2. Fig. S2 The relative abundance of the microbial genes involved in the organismal system in CACB of Pediculus humanus capitis at level 2 in KEGG pathway annotation. Comparisons shown are descriptive; statistical significance was not tested. [file 13071_2026_7471_MOESM2_ESM.tif]
